# Bone morphogenetic protein receptor signal transduction in human disease

**DOI:** 10.1002/path.5170

**Published:** 2018-11-27

**Authors:** Maria Catalina Gomez‐Puerto, Prasanna Vasudevan Iyengar, Amaya García de Vinuesa, Peter ten Dijke, Gonzalo Sanchez‐Duffhues

**Affiliations:** ^1^ Department of Cell and Chemical Biology and Oncode Institute Leiden University Medical Center Leiden The Netherlands

**Keywords:** activin, BMP, TGF‐β, bone, cancer, cardiovascular, hematopoiesis, heterotopic ossification, acromesomelic dysplasia, hemorrhagic telangiectasia, pulmonary arterial hypertension, brachydactyly, fibrodysplasia ossificans progressiva, juvenile polyposis syndrome

## Abstract

Bone morphogenetic proteins (BMPs) are secreted cytokines that were initially discovered on the basis of their ability to induce bone. Several decades of research have now established that these proteins function in a large variety of physiopathological processes. There are about 15 BMP family members, which signal *via* three transmembrane type II receptors and four transmembrane type I receptors. Mechanistically, BMP binding leads to phosphorylation of the type I receptor by the type II receptor. This activated heteromeric complex triggers intracellular signaling that is initiated by phosphorylation of receptor‐regulated SMAD1, 5, and 8 (also termed R‐SMADs). Activated R‐SMADs form heteromeric complexes with SMAD4, which engage in specific transcriptional responses. There is convergence along the signaling pathway and, besides the canonical SMAD pathway, BMP‐receptor activation can also induce non‐SMAD signaling. Each step in the pathway is fine‐tuned by positive and negative regulation and crosstalk with other signaling pathways. For example, ligand bioavailability for the receptor can be regulated by ligand‐binding proteins that sequester the ligand from interacting with receptors. Accessory co‐receptors, also known as BMP type III receptors, lack intrinsic enzymatic activity but enhance BMP signaling by presenting ligands to receptors. In this review, we discuss the role of BMP receptor signaling and how corruption of this pathway contributes to cardiovascular and musculoskeletal diseases and cancer. We describe pharmacological tools to interrogate the function of BMP receptor signaling in specific biological processes and focus on how these agents can be used as drugs to inhibit or activate the function of the receptor, thereby normalizing dysregulated BMP signaling. © 2018 The Authors. *The Journal of Pathology* published by John Wiley & Sons Ltd on behalf of Pathological Society of Great Britain and Ireland.

## Introduction

Bone morphogenetic proteins (BMPs) were initially isolated from demineralized bone matrix through their ability to induce bone at ectopic sites in rodents 1. In the late 1980s, the BMPs were purified to homogeneity and PCR primers were designed from the partial amino acid sequences that led to the cloning of cDNAs encoding several different BMPs 2. BMPs were found to be members of the transforming growth factor‐β (TGF‐β) family, which besides the TGF‐βs, also includes the activins. From the 33 genes encoding members of the TGF‐β family, about 15 structurally related BMPs have been identified 3. The most extensively investigated are BMP2, 4, 6, 7, 9, and 10. Like TGF‐β cytokines, BMPs are multifunctional proteins whose activities are highly dependent on cellular context 4. BMPs have pivotal roles in early and late embryonic development and in maintaining homeostasis in a plethora of tissues and organs.

**Figure 1:**
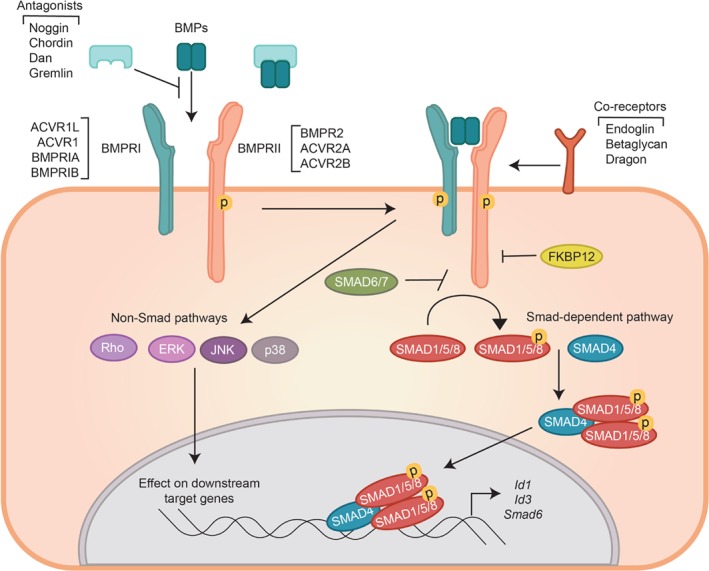
BMPR signaling. BMPs signal via complexes of type I (BMPRIs) and type II (BMPRIIs) transmembrane kinase receptors. The type II receptor, a constitutively active kinase (shown in the figure as phosphorylated), phosphorylates (specified as P) and activates the type I receptor, upon which intracellular canonical signaling is initiated by phosphorylation of receptor‐regulated R‐SMAD1, 5, and 8. Activated R‐SMADs partner with SMAD4 to transcriptionally regulate expression of specific target genes. BMPR activation can also induce non‐SMAD signaling, e.g. by activating p38 and JNK MAP kinases and small GTPases such as Rho and Rac. Each step in the pathway is fine‐tuned by positive and negative regulation. For example, ligand bioavailability for the receptor can be regulated by ligand‐binding proteins, also called antagonists, which sequester the ligand from interacting with receptors. BMP type III receptors or accessory co‐receptors can enhance BMP signaling by presenting ligands to type I and type II receptors.

In common with the TGF‐β cytokines, BMPs induce specific cellular responses through their interaction with type I and type II cell surface receptors, which activates the serine/threonine kinase activity of the receptors 5. Three distinct BMP type II receptors (BMPRIIs) and four BMP type I receptors (BMPRIs), also termed activin receptor‐like kinases (ALKs), have been characterized. Upon BMP‐induced heteromeric complex formation of specific sets of type I and type II receptors, the intracellular domains interact and the type II receptor kinase trans‐phosphorylates the type I receptor, leading to its activation. The extracellular signal is thereby transduced across the membrane and intracellular signaling initiated by phosphorylation of specific intracellular signaling proteins, including the SMAD proteins that are key transcription effectors (Figure 1). Each step of the BMP signaling pathway is controlled by positive and negative modulators allowing for signal integration with other signaling pathways 6.

Here we review the role of BMPR signaling in human diseases. Since the BMPRs share extracellular and intracellular domains that are druggable, we describe specific pharmacological agents that are available to elucidate BMPR functions in specific biological processes, and how they are/may be used to target BMP signaling in a therapeutic context. In particular, we highlight those genetic alterations of BMPRs that have been linked with diseases. We refer the reader to recent reviews for the roles of BMPRs in hematopoiesis and hematological diseases 7, reproductive tract and gynecological pathologies 8, immune regulation, autoimmunity and infection 9, and nervous system and neurodegenerative diseases 10.

## BMPR signaling

BMPs are synthesized as large pre‐pro‐precursor proteins. Each unprocessed BMP contains an N‐terminal signal peptide connected to a pro‐domain and a mature bioactive region at the carboxy terminus 3. The pro‐BMP is cleaved by a furin type protease, liberating the mature BMP containing a noncovalently attached pro‐domain. In contrast to TGF‐β (where these pro‐domains confer latency), the pro‐domains of BMPs can usually be displaced easily by receptor binding. BMPs are secreted as homo‐ and heterodimers 3. Recent studies have revealed that heterodimers can have more potent or novel functions compared to their homodimer counterparts 11, 12. In particular, BMP9 and BMP10 can heterodimerize and this heterodimer is responsible for most of the biological BMP activity found in plasma 13. BMPs mostly act in an autocrine or paracrine manner, however, some BMPs, such as BMP6, BMP9, and BMP10 13, 14 circulate in the blood and can thus act at a distance. Many BMP binding factors that interfere with BMPR binding have been identified. Examples are Noggin and Chordin, and DAN and Gremlin, which control local BMP bioavailability 15 (Figure 1).

BMPs bind to selective combinations of BMPRIs and BMPRIIs 3. BMPs are dimers and can interact with heteromeric complexes of two BMPRIIs and two BMPRIs. Whereas BMPR‐II is selective for BMPs, ActRIIA, and ActRIIB can interact with BMPs and activins. There are four BMPRIs: ALK1, ALK2 (also termed ActRI), ALK3 (also termed BMPRIA), and ALK6 (also termed BMPRIB). BMP2 and BMP4 bind more strongly to ALK3 and ALK6, BMP6, and BMP7 bind most strongly to ALK2, and BMP9 and BMP10 are high affinity ligands for ALK1 but can also bind to ALK2 3. Both receptor types share high structural and sequence similarity; they are both single pass transmembrane proteins composed of extracellular domains that are rich in cysteines and intracellular regions which harbor the kinase domain. Type II receptors, and in particular BMPR‐II, have a longer carboxy tail when compared to type I receptors. Type I receptors have a juxtamembrane region that is rich in glycine and serine residues; termed GS‐domain, these are important for activation (see below). Unlike TGF‐β and activin, that do not interact directly with their corresponding type I receptors, BMPs can bind to type I and type II alone, although the binding affinity is greatly enhanced when BMPs interact with both receptors 3. In addition, BMP accessory co‐receptors (also termed BMP type III or auxiliary receptors) have been identified, which lack an intracellular kinase activity but can regulate access of ligands to signaling receptors and/or regulate signaling specificity. Examples include the transmembrane proteins endoglin and betaglycan and GPI‐linked dragon family members (also known as repulsion guidance molecules, RGMs). Co‐receptors can share multiple different ligands, and thereby increase the possibilities for signal integration. Moreover, co‐receptors can be shed from the plasma membrane and thereby regulate BMP bioavailability systemically or exert BMP‐independent functions 16 (Figure 1).

Similarly to other TGF‐β family members, BMPRI is a substrate for BMPRII. BMPRII phosphorylates BMPRI on specific serine and threonine residues in the GS‐domain 3. The binding site of the negative regulator FKBP12 is close to this GS domain and upon binding, FKBP12 shields the serine and threonine residues from being phosphorylated by the type II kinase. Therefore, FKBP12 binding to BMPRI creates a threshold for activation, so that when type I and type II meet at the cell surface in the absence of ligand, an intracellular signal is not immediately initiated 17 (Figure 1). Upon BMPRI activation, this receptor initiates intracellular signaling by activating receptor‐regulated (R) – SMADs, i.e. SMAD1, SMAD5, and SMAD8. This is different from TGF‐β cytokines and activins, which induce the phosphorylation of R‐SMAD2 and SMAD3 in most cells 3. Phospho‐Smad specific antibodies that recognize phospho (p)‐SMAD1/5/8 and p‐SMAD2 have been generated and are often used to measure the degree of receptor activation in cells and tissues 18. Activated R‐SMADs form heteromeric complexes with the common mediator (Co)‐SMAD4 to regulate specific gene transcriptional responses through cooperation with other transcription factors, transcriptional co‐activators and repressors. R‐SMADs and SMAD4 bind to GC‐rich and CGTA containing DNA sequences, respectively. An established target gene for BMPs is *ID1*. BMP‐SMAD responsive elements were identified in this gene promotor and subsequently cloned in tandem to generate a powerful BMP‐SMAD‐responsive transcription reporter, the so‐called BRE‐luc reporter 19. Besides the canonical SMAD pathway, BMPR activation can also induce non‐SMAD signaling. Non‐SMAD signaling is characterized by intracellular activation of p38 and JNK MAP kinases and small GTPases such as Rho and Rac 20 (Figure 1).

**Figure 2:**
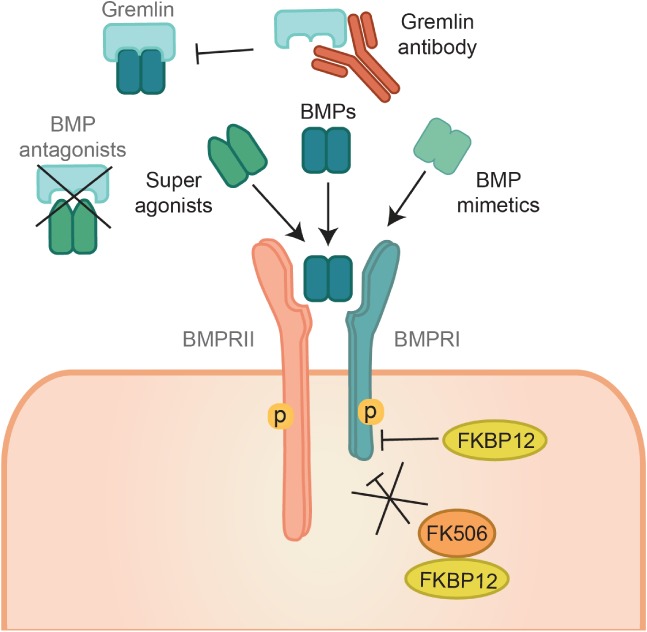
BMPR agonists. Several pharmacological agents have been developed to enhance BMPR signaling. Among them, small molecule activators of BMP signaling or BMP mimetics have been used. Furthermore, mutant BMPs that have super agonistic activity by being defective in interacting with secreted antagonists have also been engineered. In addition, neutralizing antibodies have been developed, interfering with secreted ligand‐binding proteins such as Gremlin and thereby promoting BMPR signaling. Utilization of FK506 to inhibit the interaction of the negative regulator FKBP12 with BMPRI is another approach to activate BMPR signaling.

**Figure 3:**
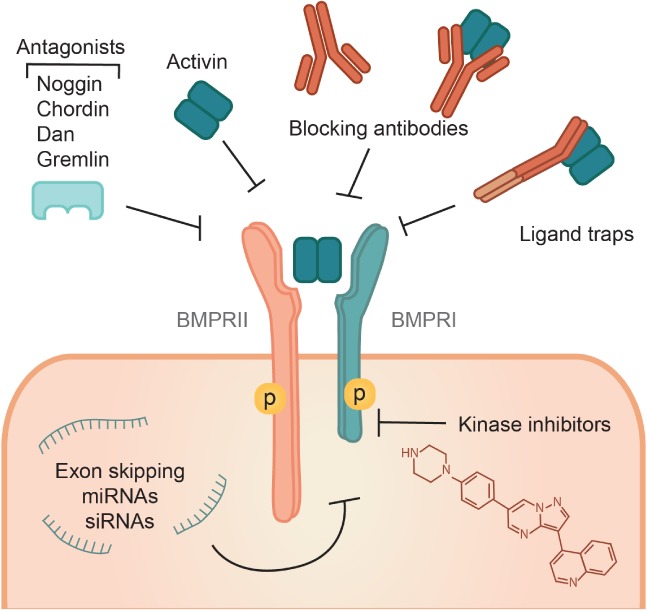
BMPR inhibitors. BMPR signaling can be inhibited through various pharmacological agents. One example are mutant BMPs, engineered to bind to but lacking the ability to activate the receptor, thereby blocking the binding of wild type BMPs to the receptor. Neutralizing antibodies that interfere with ligand–receptor interactions by recognizing either ligands or receptors have been also developed. Moreover, ecto‐domains of receptors (ligand traps) are used to sequester BMPs from binding to their receptors; functioning in a way similar to the natural secreted antagonists such as Noggin. Furthermore, small molecular kinase inhibitors have been developed that selectively interfere with BMPRI kinase activity. In addition, miRNAs, siRNAs or exon skipping have also been used as therapeutic tools to inhibit BMPR signaling.

A number of pharmacological agents have been developed to modulate BMPR signaling (Figures 2 and 3). For example, deletion mutants and folding variants of mature BMP4 have been used to prevent the binding of wild type BMP to the receptor while inhibiting ossification 21. BMPs that cannot interact with secreted antagonists have been also generated. This is the case of engineered BMP2 and BMP7 that are resistant to Noggin inhibition 22. Furthermore, neutralizing antibodies that affect ligand–receptor interactions by binding to ligands have been produced 23 (Figure 3). Another strategy has been the development of antibodies that bind to secreted ligand‐binding proteins thereby promoting BMP receptor signaling 24 (Figure 2). Moreover, ecto‐domains of receptors have been used to sequester BMPs thus preventing their binding to their receptors 25. In this way, they function similarly to natural secreted antagonists such as Noggin (Figure 3). Antibodies against the secreted antagonists were found to stimulate BMPR signaling 24 (Figure 2). Furthermore, BMPRs have intrinsic serine/threonine kinase activity, which has allowed the development of small molecular kinase inhibitors that selectively interfere with BMPRI kinase activity 26 (Figure 3). An alternative approach is the use of statins 27 and low intensity pulsed ultrasound 28 that can activate BMPR signaling by promoting BMP expression. Utilization of FK506 to inhibit the interaction of the negative regulator FKBP12 with BMPRI is another strategy 29 (Figure 2). Such modalities, in combination with animal models, have been used to determine the role of BMPR signaling in disease and have been explored as potential therapies for a number of pathologies, including cardiovascular and skeletal diseases, and cancer.

## BMPRs in cardiovascular disease

BMPRs are critical regulators of normal cardiovascular structure and function 30. Defects in cardiac development due to BMPR signaling disruption may lead to several congenital heart abnormalities. The cardiac precursor zone in the developing vertebrate embryo is shaped by an interplay between BMP, fibroblast growth factor (FGF) and Wnt signaling 31. This involves the differentiation of pluripotent and embryonic stem cells into the cardiac lineage, where BMPs play an important role. For an extensive review of BMPs and cardiomyocyte differentiation please refer to 32, 33. Septation of the outflow tract and valve maturation are also regulated by BMPs 34. In particular, BMP2 signaling has been identified as an inducer of epithelial‐to‐mesenchymal transition (EMT) in the endocardial cushions (a subset of cells found in the developing heart tube that are indispensable for valve and septa formation) 35, 36. Furthermore, conditional deletion of *Bmpr1a* (encoding ALK3) in the myocardium results in abnormal atrioventricular (AV) cushion and septal morphogenesis 37. In the developing mouse heart, ALK2 is found to mediate atrioventricular cushion transformation 38. This receptor has been associated with congenital heart defects 39, 40 and abnormal ALK2 signaling leads to the development of a bicuspid aortic valve phenotype 41.

BMPRs are key factors maintaining adult cardiovascular homeostasis and function. Here we first discuss hereditary hemorrhagic telangiectasia (HHT) and pulmonary arterial hypertension (PAH, MIM: 178600), which are two cardiovascular diseases caused by (epi)genetic alterations of BMPRs 30. HHT is a rare autosomal dominant genetic disorder leading to vascular malformations that result in direct connections between arteries and veins. The disease is characterized by mucocutaneous telangiectases and arteriovenous malformations of the gastrointestinal tract, liver, lung, and brain 42, 43. The most prevalent symptom of HHT is nosebleeds, which radically affects the quality of life of the patient. There are five subtypes of HHT: HHT type 1, 2, 3, and 4, and Juvenile Polyposis/HHT. Mutations in *ENG* (encoding Endoglin), *ACVRL1 (*encoding ALK1) and *SMAD4* cause HHT1 (MIM: 187300), HHT2 (MIM: 600376), and the combined Juvenile Polyposis/HHT (MIM: 175050) syndrome, respectively 44. While mutations in *ENG* and *ACVRL1* represent 80–85% of HHT cases, only 2% of HHT is caused by mutations in *SMAD4* 42. To date, no causative mutations have been identified in patients with HHT3 (MIM: 601101) and HHT4 (MIM: 610655). *GDF2* (encoding BMP9) mutations that negatively affect protein processing and/or function have been associated with a vascular syndrome that phenotypically resembles HHT and is now referred to as HHT5 (MIM: 615506) 45. Homozygous null *Eng* or *Acvrl1* mutations compromise angiogenesis and heart development leading to embryonic lethality in mice 46. Mice with heterozygous inactivating mutations of these genes exhibit particular vascular phenotypes which do not recapitulate the human disease completely. Therefore, conditional knockout mice, which recapitulate the specific arteriovenous malformations observed in patients, represent the most accepted animal models to study HHT 42, 46. Current therapies for HHT rely on inhibiting angiogenesis by means of Bevacizumab (VEGF inhibitor) 47 or by increasing clotting through the use of Thalidomide or antifibrinolytics 48. However, present treatments cause numerous side effects and only provide temporary symptomatic relief. To alleviate these problems, future therapies for HHT may focus on enhancing endoglin and ALK1 stability/expression or function (Table 1).

**Table 1:** Examples of diseases related to BMPRs and/or pharmacological agents based on BMPRs

Disease	Receptor*	(Experimental) Therapy†	References
Cardiovascular			
HHT1, HHT2	ENDOnitric oxide GLIN, ALK1	Tranexamic acid (NCT01031992)	49, 50, 51, 52
PAH	BMPRII, ALK1, ALK6	Tacrolimus /FK506 (NCT01647945), BMP9, Ataluren/PTC124, ActRIIA‐IgG1Fc‡ (NCT03496207), Gremlin‐1 ab	24, 29, 53, 54
Aortic valve development	ALK2		41
Left–right axis malformations	ActRIIB		55
Vascular calcification		LDN‐193189, ActRIIA‐Fc, ALK3‐Fc, Tacrolimus/FK506(NCT01612299), Dipyridamole (NCT00767663)	56, 57
Musculoskeletal			
Sarcopenia, cachexia, Duchenne muscular dystrophy		ActRII‐Fc, ActRII ab, Follistatin (NCT01519349)	
Fracture healing		OP‐1/BMP7(NCT00679328)	
Osteoarthritis		OP‐1/BMP7 (NCT01111045)	
Muscular dystrophy		ActRIIA‐Fc, ActrRIIB‐Fc	58
Fibrodysplasia ossificans progressiva	ALK2	LDN‐193189, LDN‐212854/ALK2 kinase inhibitor, activin ab, ActRIIA‐Fc, Dipyridamole	59, 60, 61, 62
Type 2 brachydactyly	ALK6		
Acromesomelic dysplasia	ALK6		
Osteoporosis		ActRIIA‐Fc, ALK3‐Fc,	63, 64, 65
Cancer		
Diffuse intrinsic pontine glioma	ALK2	LDN‐212854/ALK2 inhibitor, LDN‐214117, LDN‐193189,TP‐0184, TP‐0184 (NCT03429218, Phase 1 solid tumors)	66, 67, 68
Juvenile polyposis	ALK3		69, 70
Pancreatic cancer	ActRIIB, ALK6		71
Colon cancer	BMPRII, ActRII		72
Tumor angiogenesis	ALK1, endoglin	PF‐03446962/anti‐hALK1 ab, (NCT01620970, NCT00557856) ALK1‐Fc/Dalantercept/ACE‐041, Tracon/ TRC105 /endoglin ab (NCT02979899, NCT01381861, NCT02664961, NCT02560779, NCT01564914, NCT01332721, NCT01975519, NCT01806064, NCT02429843)	73, 74
Breast cancer	BMPR1A, BMPR2	LDN‐193189	75
Other diseases			
Alzheimer disease		OP‐1/BMP7(NCT02547818)	
Pierre Robin	ALK3		76
Juvenile hemochromatosis	RGMc/Hemojuvilin (co‐receptor for bone morphogenetic proteins)		77, 78
Anemia, defective erythropoiesis, beta thalassemia		ActRIIA‐IgG1Fc‡ (NCT00931606), LDN‐212854 (ALK2 inhibitor), or HJV‐Fc	79, 80
Myelodysplastic syndrome		ActRIIA‐IgG1Fc‡ (NCT01736683)	81
Myelofibrosis		ActRIIA‐IgG1Fc‡ (NCT01712308)	82

* Specific BMPR genes that have been found mutated in particular diseases are indicated. For some diseases no BMPR mutations are found.

† Therapies or experimental therapies that modulate the receptor expression or BMP signaling are indicated. The ClinicalTrials.gov identifier is also specified.

‡ ActRIIA‐IgG1Fc is also known as sotatercept or ACE‐011.

PAH is a chronic disease characterized by an increase in mean pulmonary arterial pressure (greater than 25 mmHg at rest), pulmonary capillary wedge pressure (15 mmHg) and pulmonary vascular resistance (greater than 3 Wood Units) 83. It is further characterized by increased endothelial cell proliferation and vascular smooth muscle cell hypertrophy resulting in progressive occlusion of the artery lumen. As a consequence, patients develop right ventricular dysfunction resulting in shortness of breath. In the absence of lung transplantation, PAH leads to right heart failure and death. More than 70% of patients with familial PAH and 20% of patients with idiopathic PAH have heterozygous mutations that compromise *BMPR2* function. These mutations target sequences that encode the ligand‐binding and kinase domain and the long cytoplasmic tail 84. Patients with PAH and *BMPR2* mutations exhibit a more severe disease and are at increased risk of death compared to those without *BMPR2* mutations 85. However, the incomplete penetrance of *BMPR2* mutations (20–30%) suggests that other genetic and environmental factors contribute to the disease. Infections, toxic exposure, inflammation 86, or alterations in estrogen metabolism 87, 88, have all been described and some were reported to downregulate BMPRII expression 89. Besides mutations in the *BMPR2* gene, mutations in genes encoding for other BMP signaling components (such as *GDF‐2* 90, 91, *ACVRL1* 92, *ENDOGLIN* 93, and *SMAD8* 53, 94) are also associated with PAH development. The BMP/SMAD signaling axis is perturbed in monocrotaline (MCT)‐treated rats 95, one of the most broadly used models to study PAH. The authors showed a decrease in BMPR2, ALK6, and SMADs 4, 5, 6, and 8 expression in the lungs but not the kidneys of MCT‐treated rats 95. Furthermore, *Bmpr2* deficient rats generated to study PAH revealed increased endothelial‐to‐mesenchymal transition (EndMT), which was implicated in occlusive vascular remodeling 96. Current PAH therapies target prostacyclin, endothelin, and nitric oxide (NO) pathways, which are involved in vasodilation 97. High‐dose calcium channel blockers, anti‐inflammatory and anti‐proliferative drugs are also being used. Novel strategies to treat PAH patients focus on increasing BMPRII transcription or expression and on blocking BMPRII degradation. Finally, activating BMPRII/SMAD signaling by FK506 98 or enhancing BMP signaling by exogenous recombinant BMP9 54, 99 are interesting approaches that have shown promising results in PAH animal models, but could prove difficult to translate into treatments for patients (Table 1).

Lastly, in addition to the aforementioned vascular diseases that have been linked to genetic mutations in BMPRs, aberrant expression of BMPRs has also been implicated in atherosclerosis 100, vascular calcification, and anemia 101.

## BMPRs in musculoskeletal disease

BMPs tightly modulate bone homeostasis by targeting cells with osteogenic and chondrogenic potential, as well as osteoclasts and vascular cells (reviewed in 102). Due to their pleiotropic roles, complete ablation of BMPRs often leads to embryonic lethality. To dissect the specific *in vivo* roles of these receptors, conditional knock‐outs in defined cell types have been generated, showing that disturbed BMPR expression usually leads to bone and cartilage disorders in animal models, including low 103 and high bone mass 104, 105, heterotopic ossification (HO) 106 and defective cartilage formation 107, 108, 109, 110, 111.

A number of genetic bone disorders in which BMPRs are mutated have been described. One example is Acromesomelic dysplasia, a particular form of dwarfism that affects the bones of the hands and feet (*acromelia*) as well as the forearms and lower legs (*mesomelia*). There are several forms of this condition. Acromesomelic dysplasia Grebe type (AMDG, MIM: 200700) 112 and Demirhan type (AMDD, MIM: 609441) 113 are associated with biallelic loss of activity mutations in *BMPR1B* (encoding the BMP type I receptor ALK6). Brachydactyly type B (BDB1, MIM: 113000) is a very rare skeletal condition characterized by shortening of the middle phalanges and absent or rudimentary terminal phalanges. BDB1 has been associated to inactivating mutations in the *ROR2* gene 114, 115. Interestingly, the receptor tyrosine kinase ROR2 has been shown to physically interact with ALK6 in a very specific manner 116, 117. BDB1 shares most of its clinical features with Brachydactyly type A2 (BDA2, MIM: 112600), characterized by shortening of the middle phalanx of the index finger and abnormal second toe development. Additionally, inactivating mutations in the *GDF5* and *BMP2* genes have been described 118, 119. *BMPR1B* was found to be mutated in either the kinase domain (I200K) or the GS domain (R486W) 120. Using micromass cell cultures and chick limbs assays, overexpression of either I200K or R486W ALK6 caused reduced cartilage differentiation and bone formation. Interestingly, although both mutant BMPRs retained their usual localization at the membrane, only the mutation in the kinase domain resulted in decreased kinase activity 120.

Fibrodysplasia ossificans progressiva (FOP, MIM: 135100) is the most devastating congenital disorder involving HO, that is, endochondral bone formation at extra‐skeletal sites. FOP is characterized by malformation of the big toes at birth and episodic HO in tendons, fascia, ligaments, and muscle 121, which progressively results in the patients being wheelchair‐bound by the third or fourth decade of life. It is noteworthy that inflammation has been identified as a common trigger of HO in FOP 122. The *ACVR1* gene encodes ALK2 and a point mutation in the *ACVR1* gene (c.617G>A) leading to illicit activation of ALK2 was identified more than a decade ago in nearly 95% of all studied cases of FOP 123. The mutation, which effects the cytosolic GS domain of the receptor, causes an amino acid substitution R206H that was shown to constitutively activate the receptor by interfering with binding of the negative regulator FKBP12 124 and/or enhancing 125 the response to certain BMP ligands. This led to the development of BMPRI kinase inhibitors as a possible treatment for FOP (Table 1). A recent discovery showed that activin A is capable of inducing ALK2 downstream signaling in FOP mutant cells 59, 126. Although activins are known to interact with ALK2, they do not engage the receptor directly and instead compete with osteogenic BMPs 127 **.** It is noteworthy that other *ACVR1* mutations found in a minority of FOP patients also induce ALK2 downstream signaling in response to activin A 126, 128. How intracellular mutations alter the activation of ALK2 upon extracellular binding of activin A remains to be unveiled. Receptor binding affinity assays showed increased binding of iodinidated activin A to ALK2 R206H in a receptor complex including ActRIIA or ActRIIB 126. This suggests that the mutant ALK2 receptor exhibits a higher affinity for activin A than the wild type receptor. Accordingly, sequestration of circulating activins by an ActRIIa‐Fc ligand trap or a specific activin A antibody prevented HO in an animal model of FOP. This mechanism might specifically target bone progenitor cells in FOP. In this regard, a recent publication has identified one tendon‐derived Scx^+^ cell population and one muscle resident Mx1^+^ cell population, which could specifically drive the formation of heterotopic bone either in tendons or in muscle 129.

Myostatin, which like TGF‐β induces R‐SMAD2/3 phosphorylation, is best known for its ability to negatively regulate muscle mass and muscle fiber size 130. This process is counterposed by SMAD1/5 signaling, which induces protein synthesis in muscle (reviewed in 131). Accordingly, intramuscular AAV‐mediated overexpression of a constitutive form of ALK3 resulted in increased fiber size, maximal force and muscle mass 131, whereas overexpression of the BMP inhibitor Noggin, or injection of short hairpin RNAs (shRNAs) targeting SMAD1 or SMAD5, led to reduced myofiber size and muscle atrophy. Another group independently showed that over‐activation of BMP signaling in muscle by ectopic expression of either BMP7 or a constitutively active ALK3 prevented muscular atrophy. Furthermore, the authors showed that BMP‐induced signaling counteracts the histone deacetylase (HDAC)4‐myogenin axis that normally contributes to muscle atrophy in denervated muscles 132. Mice in which *Bmpr1a* (encoding ALK3) was selectively ablated in Myf5^+^ or MyoD^+^ quiescent satellite cells revealed more fat accumulation in muscles when compared to controls 133. The authors demonstrated that ALK3 expression in myo‐endothelial Myf5^+^ cells is necessary for these cells to support the activity of adipogenic progenitors within the muscle. Taken together, accumulating knowledge suggests that bone and muscle homeostasis is tightly controlled by BMP signaling and aberrant BMPR function may lead to bone and skeletal muscle disorders.

## BMPRs in cancer

Several studies have demonstrated a strong link between mutations of certain BMPRs and the progression of specific cancers. Moreover, aberrant expression of these receptors has been correlated with a poorer prognosis for cancer patients. For instance, in Juvenile polyposis syndrome (JPS, MIM: 174900), an autosomal dominant inherited disorder, mutations in *BMPR1A* have been associated with the development of gastrointestinal cancers 134. A study of several families with JPS identified nonsense inactivating mutations giving rise to ALK3 receptors which lack the intracellular kinase domain 135. Other studies confirmed the overall prevalence of *BMPR1A* mutations in JPS patients, revealing point mutations as well as large deletions of the gene in approximately 23% of the JPS patients studied 136.

Mutations in *ACVR1* have been described in pediatric cases of diffuse intrinsic pontine gliomas (DIPGs) 137, 138. These gain of function mutations are similar to those found in FOP (R206H, R258G/S, G328V/E/R/W, and G356D) 128. DIPG is a highly infiltrative and fatal form of cancer that is usually inoperable due to the location of the tumor. Frequent somatic mutations in histone H3 have been well‐documented in these patients. Moreover, co‐occurrence of *ACVR1* mutations have been shown to cause an overall increase in *ID1* and *ID2* levels. Also, through *in vitro* experiments, ALK2 mutations have been shown to enhance cell proliferation 139. Surprisingly, although benign osteochondromas have been reported in the majority of FOP patients 140, lack of oncogenic predisposition in FOP patients with identical mutations in *ACVR1* suggests that the mutant *ACVR1* favors tumor development initiated by Histone H3 disruptions. In sporadic colorectal cancer with a microsatellite unstable phenotype, the *BMPR2* 3′ untranslated region is frequently mutated 72. This often coincides with mutations in *ActRII* and *TGFBR2* leading to a loss of TGF‐β receptor signaling. In pancreatic cancer, reduced expression of ALK3 was found to be associated with a poor prognosis 141. In such cases, changes in receptor expression could be used as prognostic biomarkers.

Besides these genetic changes, misexpression of BMPRs can also contribute to cancer progression. For example, interference with the function of endoglin and ALK1 co‐receptors, which are expressed on proliferating endothelial cells, has been shown to inhibit tumor angiogenesis. The latter is needed for the growth of tumors beyond a few cm^3^ in size and also for efficient metastasis. Targeting endoglin with a neutralizing antibody is currently being tested in clinical trials, which are showing variable outcomes (Table 1) 142, 143, 144. The use of Tracon/TRC105 has been tested either alone or in combination with other anti‐angiogenic drugs to inhibit the formation of new blood vessels in solid tumors. Inhibition of ALK1 function using a neutralizing antibody or an ALK1‐Fc ligand trap (dalatracept) resulted in a minor therapeutic response as single agent in phase 2 clinical trials in cancer patients (Table 1) 145, 146, 147.

## Conclusions and perspectives

BMPs stimulate multiple signaling pathways in a large variety of cell types via three type II and four type I receptors. The inactivation or over‐activation of these receptors by genetic alterations or misexpression can lead to specific cardiovascular, musculo‐skeletal diseases and cancer: (1) HHT is linked to mutations in the co‐receptor endoglin and ALK1; (2) primary PAH is associated with mutations in BMPR2 or its reduced expression; (3) gain of function mutations in ALK2 result in FOP; and (4) loss of function mutations in ALK3 are associated with JPS.

BMPR agonists or antagonists have been used to investigate the role of BMPR function in various pathophysiological processes. New functions for BMPRs continue to be discovered beyond the role as an inducer of bone. Moreover, BMPR agonists or antagonists can be used as pharmacological agents to treat diseases characterized by changes in BMPR activity. Nevertheless, because of the pleiotropic roles of BMPRs in different cell types and diseases, it is important to consider possible off‐target effects including undesirable actions of receptor complexes or aberrant immune responses. However, it is likely that the advantages of targeting these pathways will outweigh the problems and such strategies might be used to inhibit tumor angiogenesis by antagonizing endoglin or ALK1 function and also to increase muscle mass and alleviate anemia using ActRII‐Fc ligand traps. While potent effects have been reported in animal disease models, translation towards human patients has been disappointing thus far, with no significant effects reported to date 99. An obvious reason for this failure is that animal models do not recapitulate the human disease completely, or that these BMPR targeting agents are only relevant for a particular subset of patients. Another explanation is that the drugs are tested as single agents on patients that have not been carefully screened to determine if they might benefit from the treatment.

The use of CRISPR‐Cas9 may allow the development of better animal models that more closely mimic human diseases. Utilization of patient‐derived models using human (induced pluripotent cell‐derived) heterotypic organoid cell cultures grown in 3D within an appropriate extracellular matrix 148 may enable a more thorough preclinical assessment of the therapeutic potential of BMPR modulators either as single agents or in combination with other molecules that synergistically enhance their activities. Finally, the efficacy of treatments could be enhanced, and off‐target effects reduced, by more precise pharmacological dosing regimens, e.g. by putting patients on so‐called drug holidays 149 and/or by using precision delivery devices 150.

## Author contributions statement

MCGP, PVI, PTD, and GSD performed the literature search. AGV designed and generated the figures. MCGP, PVI, PTD, and GSD wrote the manuscript. All authors were involved in the manuscript and all authors approved the final version.
